# Vitamin D and Acute Kidney Injury: A Two-Way Causality Relation and a Predictive, Prognostic, and Therapeutic Role of Vitamin D

**DOI:** 10.3389/fnut.2020.630951

**Published:** 2021-03-04

**Authors:** Spyridon Graidis, Theodosios S. Papavramidis, Maria Papaioannou

**Affiliations:** ^1^Laboratory of Biological Chemistry, Faculty of Health Sciences, School of Medicine, Aristotle University of Thessaloniki, Thessaloniki, Greece; ^2^1st Propedeutic Department of Surgery, Faculty of Health Sciences, School of Medicine, AHEPA University Hospital, Aristotle University of Thessaloniki, Thessaloniki, Greece

**Keywords:** vitamin D, acute kidney injury, pathogenesis, biomarker, prediction, prognosis, therapeutic agent

## Abstract

**Background:** Acute kidney injury (AKI) constitutes a multi-factorially caused condition, which significantly affects kidney function and can lead to elevated risk of morbidity and mortality. Given the rising scientific evidence regarding vitamin D's (VitD's) multisystemic role, the connection between AKI and VitD is currently being studied, and the complex relation between them has started to be unraveled.

**Methods:** A systematic review had been conducted to identify the pathogenetic relation of VitD and AKI and the potential role of VitD as a biomarker and therapeutic–renoprotective factor.

**Results:** From 792 articles, 74 articles were identified that fulfilled the inclusion criteria. Based on these articles, it has been found that not only can VitD disorders (VitD deficiency or toxicity) cause AKI but, also, AKI can lead to great disruption in the metabolism of VitD. Moreover, it has been found that VitD serves as a novel biomarker for prediction of the risk of developing AKI and for the prognosis of AKI's severity. Finally, animal models showed that VitD can both ameliorate AKI and prevent its onset, suggesting its renoprotective effect.

**Conclusion:** There is a complex two-way pathogenetic relation between VitD disorders and AKI, while, concomitantly, VitD serves as a potential novel predictive–prognostic biomarker and a treatment agent in AKI therapy.

## Introduction

Acute kidney injury (AKI), acute kidney failure (AKF), or acute renal failure (ARF) is a multi-factorially caused condition, characterized by sudden and rapid loss of kidney function. Acute kidney injury results in further systemic disorders and is related to elevated morbidity and mortality (1). There are many different definitions for AKI, characterized by different biochemical, physiological, and clinical cutoff points. The most preponderant ones are based on serum creatinine (sCr), glomerular filtration rate (GFR), and urine output (UO). According to the Kidney Disease Improving Global Guidelines (KDIGO) Clinical Practice Guidelines for Acute Kidney Injury definition, there are three stages of AKI. Stage 1 includes one of the following: (a) 1.5–1.9 times baseline sCr, (b) ≥ 0.3 mg/dl increase of baseline sCr, or (c) <0.5 ml/kg/h UO for 6–12 h; Stage 2 includes one of the following: (a) 2–2.9 times baseline sCr or (b) <0.5 ml/kg/h for ≥12 h; and Stage 3 includes one of the following: (a) 3 times baseline sCr, (b) ≥ 4.0 mg/dl increase, (c) initiation of renal replacement therapy (RRT), (d) in patients <18 years old, a decrease of eGFR <35 ml/min/1.73 m^2^, (e) <0.3 ml/kg/h for >24 h, or (f) anuria ≥12 h (2).

The pathophysiology of AKI can be classified into: (1) prerenal azotemia (disruption of kidneys' blood inflow, without cellular or structural damage), (2) intrinsic or intrarenal AKI (direct damage to kidney's structure) due to acute tubular necrosis (ATN), vascular damage, and glomerular damage, (3) interstitial AKI (damage of the interstitial tissue), or (4) postrenal AKI (obstruction of urinary tract) (3). Moreover, AKI can be induced by septic shock, cardiogenic shock, pharmaceutical administration, hepatorenal syndrome, obstructive uropathy, and hypovolemia (4).

Due the necessity to specify the causes and the risk factors inducing AKI, many efforts have been made in order to identify sensitive, specific, and cost-efficient biomarkers, which may contribute to early detection and disease prognosis. In this regard, research has been also focused on the relation between AKI and vitamin D (VitD).

Apropos of VitD, it is an important part of the physiological function of various systems. VitD is, mainly, produced in the skin *via* UV radiation–induced transformation of 7-dehydroxycholesterol (7DHC) to cholecalciferol, which then undergoes consecutive hydroxylations in the liver and kidneys, accordingly, which leads to the synthesis of 1,25(OH)_2_D (5, 6). However, 1,25(OH)2D can be synthesized into many extrarenal tissues, which possess the CYP27B1 enzyme, which is essential for the process (7). VitD's effects are classified into endocrine, paracrine, and autocrine, while the mechanism of action is both genomic [based on the action of VitD receptor (VDR) on VitD response elements (VDREs)] and non-genomic (8, 9). Briefly, VitD affects, significantly, calcium–phosphate homeostasis, leads to bone reabsorption, enhances muscular contraction and proliferation, intensifies myocardial contracture and lowers blood pressure *via* affecting the renin–angiotensin–aldosterone system (RAAS), enhances innate immunity and changes the cytokine and chemokine profile of acquired immunity from proinflammatory to anti-inflammatory, and protects against autoimmune response and cancer (10).

The aim of the present systematic review is to unravel the relationship between AKI and VitD in both patients and experimental models. We assumed that this is a two-way relationship, insinuating that on the one hand, VitD levels may play a role in AKI, and inversely, AKI induces changes in VitD levels. In this perspective, we examined whether hypo- or hypervitaminosis causes AKI and, inversely, whether AKI causes hypo- or hypervitaminosis. It was actually pointless to search for active comparators. Moreover, we examined possible implications of VitD as a predictive and prognostic marker for AKI, as well as its role in AKI treatment.

## Methods

In May 2020, a bibliographic search was conducted in PubMed, Scopus, and Embase regarding the relation between VitD and AKI. The terms employed were “vitamin D AND AKI,” “vitamin D AND ARF,” and “vitamin D AND acute kidney injury.” Additionally, a manual search of the reference lists of other studies had been conducted, to detect subsequent material that could be included in this systemic review.

Then, the articles were screened, based on:

1. Removing duplicates
2. Relativity to the subject
3. Date of publication
4. Validity of methods and conclusions

Articles that did not meet the above criteria were excluded.

## Results

**Figure 1:**
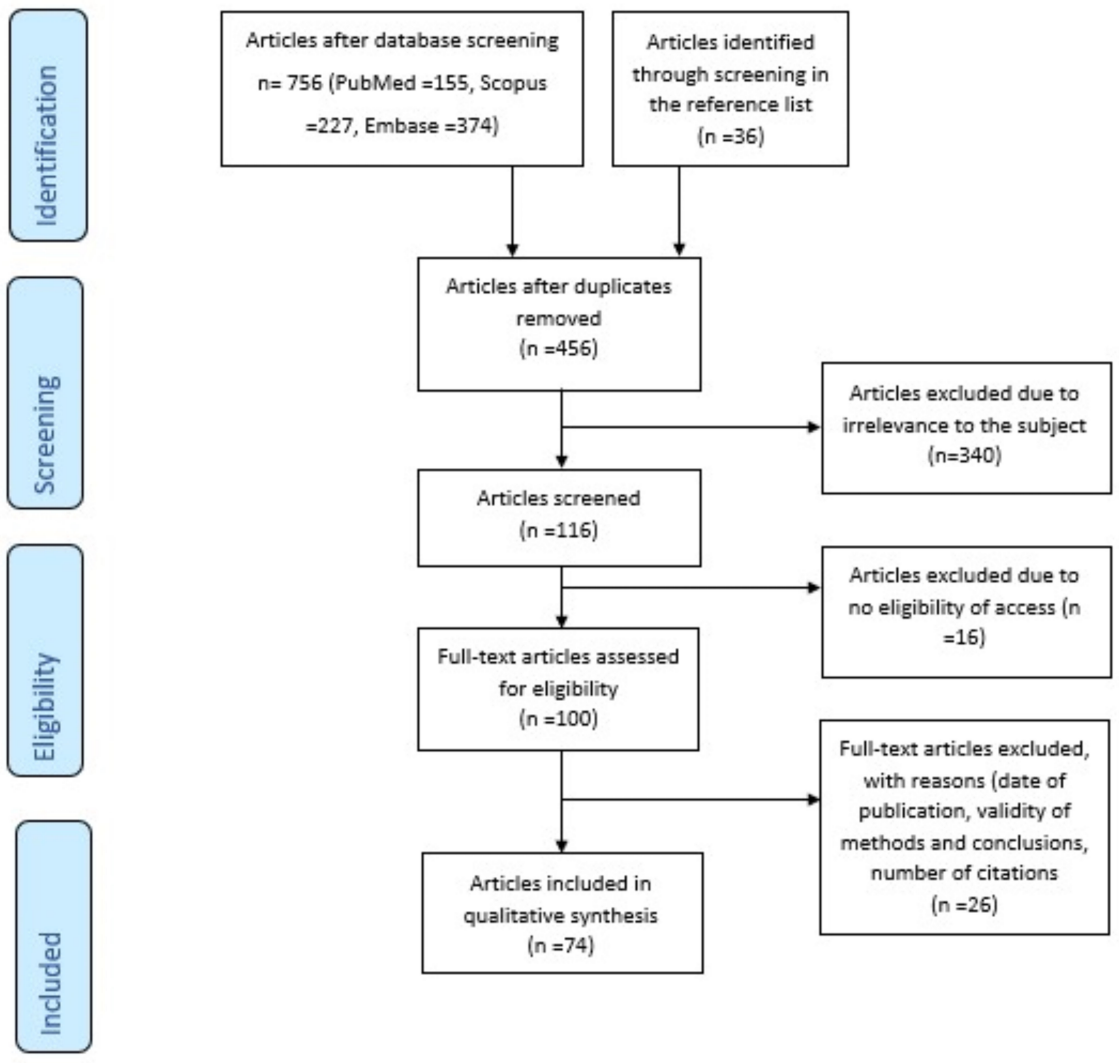
Prisma flow diagram of the systematic review's methodology: This figure clearly depicts the methodology of the identification, screening, and exclusion of articles, in order to reach the final number (*n* = 74) of articles that are relevant to the topic and fulfill the inclusion criteria.

There were 756 articles identified in the three databases. Moreover, 36 articles have been included from screening the reference lists of the articles. After the exclusion of 336 duplicates and 340 irrelevant studies, the remaining 117 articles were analyzed based on the eligibility for full access and the inclusion criteria. Finally, 74 articles matched our search criteria (Figure 1).

These articles reveal a two-way relation between VitD and AKI. Acute kidney injury can, *via* various mechanisms, lead to either D hypo- or hypervitaminosis. Moreover, both VitD deficiency and VitD toxicity can lead to the development of AKI.

Given the fact that our knowledge concerning the relationship between VitD and AKI comes from small series, cases, and experimental models, extrapolation of our results to the general population may be risky and biased. Moreover, introducing VitD in the treatment of AKI is also questionable on a clinical basis since there are no prospective randomized trials available and our knowledge is a result of animal experiments. Finally, more clinical data are available on the use of VitD levels as biomarkers concerning both the prediction and the prognosis of AKI. However, despite the fact that there are nine clinical studies on this matter, those studies are observational (either prospective or retrospective), and no data coming from randomized controlled trials are available.

## Discussion

### The Development of AKI Is Associated With VitD Disorders

Acute kidney injury is the significant depletion of renal function, affecting the normal renal enzymatic activity, and consequently disrupts VitD. Usually, the decreased kidney function causes VitD deficiency, but in some cases, VitD toxicity is also observed (Table 1).

**Table 1:** AKI as a cause of vitamin D (VitD) disruptions: AKI leads to the development of either hypovitaminosis D or hypervitaminosis D through various mechanisms.

**Condition**	**Mechanism**	**Citation**
Hypovitaminosis D	Retention and accumulation of phosphate.	(11, 12)
	Depletion of active nephrons, where the second hydroxylation occurs.	(11)
	Klotho deficiency leads to secondary increase of FGF-23 and deterioration of AKI.	(13, 14)
	Increase of FGF-23.	(13, 15)
Hypervitaminosis D	Due to calcium's diminution, the secondary hyperparathyroidism leads to induction of CYP27B1.	(6, 11)
	Rhabdomyolysis-induced mechanism.	(16)

*This table presents the mechanisms via which AKI can lead to either VitD deficiency or VitD toxicity. FGF-23, fibroblast growth factor-23; AKI, acute kidney injury; CYP27B1, cytochrome P450 family 27 subfamily B member 1 or 1α-hydroxylase*.

The progressive depletion of renal function during AKI leads to retention and accumulation of phosphate, due to the inability to excrete it. Phosphate acts as a negative regulator of 1-hydroxylase, an enzyme of 1,25(OH)_2_D synthesis, and therefore negatively contributes to VitD metabolism (11, 12). Moreover, as the final hydroxylation takes place in the kidney's active unit, the gradual loss of active nephrons leads to reduced ability of VitD synthesis (11) and to diminution of calcium's intestinal absorption. However, calcium's concentration depletion contributes to an increase of the parathyroid hormone (PTH). Due to secondary hyperparathyroidism and induction of CYP27B1, the levels of VitD can also be elevated (6, 11).

Fibroblast growth factor-23 (FGF-23), a phosphaturic factor, induces phosphaturia *via* acting on FGF-receptor (FGFR)/Klotho coreceptor complex, by (1) downregulating the luminal sodium/phosphate cotransporter in the proximal convoluted tubules, (2) suppressing kidneys' 1-hydroxylase, and (3) inducing 24-hydroxylase, which catalyzes the first step of VitD catabolism, causing hypophosphatemia and significantly lower levels of active VitD (13, 14). The soluble Klotho (sKl) can also regulate calcium and phosphate metabolism *via* an FGF-23–independent mechanism affecting the renal outer medullary potassium (ROMK) channel and the transient receptor potential vanilloid-5 (TRPV5). Moreover, sKl has a renoprotective effect *via* (1) the transforming growth factor-β1 (TGF-β1)–dependent anti-fibrotic mechanism and (2) the tumor necrosis factor (TNF)/nuclear factor-κB (NFκB)–dependent anti-inflammatory mechanism (14).

VitD deficiency can be caused by increased expression of FGF-23, which has been observed in AKI cases. Leaf et al. presented a case of a 45-year-old male hospitalized due to rhabdomyolysis and with a history of polysubstance abuse. The concurrent AKI was accompanied by elevated levels of FGF-23 and slightly decreased levels of 25(OH)D and 1,25(OH)_2_D (15). Additionally, FGF-23 can be secondarily elevated due to Klotho deficiency, which is prevalent, mostly, in chronic kidney disease (CKD) but also has been observed in AKI (13). Klotho's deficiency affects VitD levels, not only by upregulating FGF-23 but also through increasing the extent of AKI, due to depleted renoprotection, and leading, consequently, to greater VitD deficiency (14).

Moreover, Akmal et al. presented a study with patients suffering from rhabdomyolysis and AKI. The patients were initially hypocalcemic. However, during the diuretic phase, an elevation of 25(OH)D and 1,25(OH)_2_D was observed, which was greater in patients who developed hypercalcemia (16). These results suggest that the elevation of serum 1,25(OH)_2_D plays an important role in the development of hypercalcemia and that this increase may be due to extrarenal production or/and dysregulated renal production.

### VitD Deficiency Induces AKI

VitD depletion, VDR knock-out, or disruption of VitD synthesis contributes to AKI development (Table 2) by leading to upregulation of RAAS and to elevated mRNA expression of renal-vascular renin. Due to obstruction and increased levels of extracellular matrix proteins (such as collagen I and fibronectin) and proinflammatory and profibrogenic factors [such as TGF-β, connective tissue growth factor, and monocyte chemoattractant protein-1 (MCP-1)], the renal injury becomes more severe. Moreover, epithelial-to-mesenchymal transition (EMT) was observed (17). Upon angiotensin I antagonist administration, there was no difference between wild and VDR^−/−^ mice, suggesting that angiotensin II is responsible for the increased renal damage. Contrarily, when VDR agonists were administrated, proteinuria, podocytes' damage, mesangial dilation, macrophage infiltration, oxidative stress damage, proinflammatory and profibrogenic factors, and extracellular matrix protein and neutral lipid accumulation were reduced, proposing that VDR depletion worsens renal injury (17, 18).

**Table 2:** VitD deficiency induces AKI through various mechanisms.

**Condition**	**Type of model**	**Mechanism**	**Citation**
VitD deficiency	Wild-type and VDR^−^/^−^ mice	Upregulation of RAAS *via* lack of obstruction of angiotensin II receptor and mineral-corticoid receptor	(17)
	Diet-induced obesity mice	Elevation of extracellular matrix proteins and of profibrogenic and proinflammatory factors	(18)
	Wistar rats	Deterioration of already-existing renal vascular damage (ischemia/reperfusion injury induced AKI), which leads to accelerated AKI-to-CKD progression *via* increased TGF-β1 signaling and *via* decreased VDR and Klotho expression	(19–21)
	Human case	In rhabdomyolysis-induced AKI, simultaneous administration of atorvastatin and sitagliptin	(22)

*This table presents the mechanism through which VitD deficiency can lead to the development of AKI. RAAS, renin–angiotensin–aldosterone system; CKD, chronic kidney disease; TGF-β1, tumor growth factor-β1; VDR, VitD receptor*.

Moreover, VitD deficiency can exacerbate pre-existing AKI [ischemia/reperfusion injury (IRI) induced AKI] by deteriorating the renal vascular condition and it can accelerate the AKI-to-CKD progression, *via* both an increased TGF-β1 signaling and a decreased VDR and Klotho (19–21).

Buttar et al. presented a case of an 86-year-old woman with mild chronic disease and decreased levels of 25(OH)D, who developed rhabdomyolysis and secondary AKI after sitagliptin administration. The patient was on chronic atorvastatin therapy, and a possible interaction between these two drugs is proposed (22).

### VitD as a Predictive and Prognostic Biomarker

In current clinical practice, most assays estimate total 25(OH)D, which cannot distinguish the three different forms of 25(OH)D [VitD binding protein (VDBP)–bound 25(OH)D, albumin-bound 25(OH)D, and free 25(OH)D]. This approach has many limitations since there are many variables that are influenced by physiologic and pathophysiologic conditions. The VDBP's affinity is affected by both hyperlipemic conditions and the three common variants of VDBP's gene, GC1F (group-specific component-1f), GC1S, and GC2 (23). Many efforts were made at inventing new assays that can directly, validly, efficiently, affordably, and quickly estimate free 25(OH)D, rather than calculating it using already-existing multi-factorial formulas. Free 25(OH)D measurement is considered to have more benefits than total 25(OH)D. So far, the studies have only proved the benefits of free 25(OH)D (1) in cases with differences in VDBP affinity, (2) in the elderly, (3) in pregnancy to detect VitD deficiency, (4) in liver diseases, (5) in kidney disorders (AKI, CKD), (6) in acromegaly, and (7) in allergies associated with atopy and pulmonary function in asthmatic children (23, 24). However, in many studies, no significant superiority of free 25(OH)D was observed. Still, there were many limitations in some of the studies, due to sample size and due to the use of monoclonal VDBP kits in multiracial/non-Caucasian populations, which affected the outcome (23, 24).

Regarding the predictive and prognostic role of VitD and its metabolites, mostly 25(OH)D but, also, 1,25(OH)_2_D can act as novel biomarkers of AKI (Supplementary Table 1).

Braun et al. found that 25(OH)D could serve as an independent predictor of AKI since serum 25(OH)D deficiency (<15 ng/ml) and insufficiency (15–30 ng/ml) are associated with greater risk of AKI. Moreover, serum 25(OH)D can also act as independent prognostic biomarker of 30-day mortality since its deficiency and insufficiency are linked to elevated risk of 30-day death (25).

Similarly, Zapatero et al. showed that serum 25(OH)D can act as a predictor of AKI, given that patients with serum 25(OH)D <10.9 ng/ml had greater risk of AKI than VitD-sufficient patients. Moreover, lower levels of serum 25(OH)D were more common in non-survivor patients, and serum 25(OH)D acted as a prognostic biomarker of mortality (best cutoff value = 10.9 ng/ml) (26).

Leaf et al. found by using a two-multivariable-adjustment model that bioavailable 25(OH)D could serve as an independent prognostic biomarker of sepsis severity (*r* = −0.45) and mortality, while 25(OH)D can only correlate with sepsis severity (*r* = −0.42). Also, VDBP, 1,25(OH)_2_D, and FGF-23 are predictive biomarkers of AKI, while FGF-23 served as biomarker of sepsis severity too (*r* = 0.35) (27).

Sahin et al. found that VitD acts as an independent predictive biomarker of contrast-induced nephropathy (CIN)–AKI, even in multivariable models (28).

Chaykovska et al. found that neither urinary VDBP (uVDBP) nor uVDBP/sCr could predict AKI development, but they served as biomarkers of need-of-dialysis, mortality, major adverse renal events (MAREs) (only uVDBP), and non-elective hospitalization. After adjustments, the predictive value of uVDBP was confirmed and was independent of well-known CIN risk factors, such as anemia, already-existing kidney injury, heart failure, and diabetes (29).

Vicente-Vicente et al. found that uVDBP can, potentially, predict the risk of gentamicin-induced AKI, in order to prevent its manifestation, given that increased uVDBP is associated with chronic proclivity to gentamicin nephrotoxicity (30).

Rebholz et al. found that VDBP, free 25(OH)D, bioavailable 25(OH)D, total 25(OH)D, and 1,25(OH)_2_D (it is the only dependent biomarker) act as independent biomarkers of development of the ESRD stage, even after various multivariable models of adjustments (31).

Lai et al. studied the predictive and prognostic value of 1,25(OH)_2_D, 25(OH)D, and [1,25(OH)_2_D/25(OH)D] × 1,000 ratio. As for the prediction of AKI, only 1,25(OH)_2_D and [1,25(OH)_2_D/25(OH)D] × 1,000 ratio were significant, while as for the prognosis, these two biomarkers had been negatively correlated with AKI stage stratification (Risk, Injury, Failure). None of the markers could predict mortality, while 25(OH)D showed neither predictive nor prognostic effect (32).

Leaf et al. studied the prognostic value of human cathelicidin antimicrobial protein-18 (hCAP-18), free 25(OH)D and bioavailable 25(OH)D, and the predictive value of hCAP-18. As for the prognosis, hCAP-18 measured on ICU day 1 is an independent biomarker of sepsis and 90-day mortality, as had been found *via* univariate and multivariate models of adjustments. However, hCAP-18 showed no significant predictive effect regarding AKI. As for the prognostic value of free and bioavailable 25(OH)D, only the former acted as a biomarker of 90-day mortality, while the latter showed no significant prognostic value (33).

### Vitamin D in the Treatment of AKI

Many studies have been performed mainly with rats to define the potential use of VitD as treatment for AKI (Supplementary Table 2).

A dominant cause of AKI is IRI, induced by the actuation of inflammation and the increased expression of matrix metalloproteinases (MMPs). Ersan et al. studied the effect of paricalcitol on MMPs expression and, subsequently, on IRI progress. Pre-treatment with paricalcitol resulted in amelioration of IRI-AKI *via* an MMP-dependent inflammatory mechanism (34).

Hamzawy et al. studied the effect of pre-treatment with 22-oxacalcitriol (OCT) on IRI-AKI and found that it can ameliorate AKI through: (1) an anti-inflammatory mechanism *via* inhibition of Toll-like receptor-4 (TLR-4) and interferon-γ (IFN-γ), (2) a reduction of Na^+^/H^+^ exchanger-1 (NHE-1 exchanger), (3) a pro-autophagic action *via* elevating Beclin-1 expression and LC3II/LC3I ratio, (4) an anti-apoptotic action *via* elevating Bax/Bcl-2, cytochrome c and caspase-3 expression, and (5) an inhibitory action on G1 cell cycle arrest *via* reducing insulin-like growth factor-binding protein-7 (IGFBP-7) and tissue inhibitor of matrix metalloproteinases-2 (TIMP-2) expression (35).

In another study, Kapil et al. also investigated the protective role of VitD pre-treatment in IRI-AKI and showed a renoprotective effect in IRI against oxidation and lipid peroxidation mediated by peroxisome proliferator–activated receptor-γ (PPAR-γ)- (36).

Additionally, Arfian et al. studied the impact of VitD on IRI-AKI and found that its administration can mitigate AKI through reducing the inflammation and the production of myofibroblasts, *via* reducing the expression TLR-4 and MCP-1 (37).

Also, Silva Barbosa studied the effect of estrogen sulfotransferase (SULT1E1) inhibition on IRI-AKI and found that this inhibition attenuates AKI *via* elevating the VDR activation, as shown by the elevated Cyp24α1 and Ccnd1 and by the decreased Fgg expression (38). This effect has been found to be estrogen- and androgen-independent (38). Additionally, only in male mice, the liver expression of Sult1e1 has been found to be necessary for IRI-AKI development, thus proposing a tissue- and sex-specific relation between the expression of Sult1e1 and sensitivity to IRI-AKI (38).

Moreover, Lee et al. examined the effect of paracalcitol on IRI and demonstrated that it can attenuate AKI *via* an anti-inflammatory mechanism mediated by the inhibition of TLR-4 expression and the suppression of NFκB signaling, by increasing IκB in a TNF-α-dependent way (39).

Xu et al. studied the effect of pre-treatment with VitD on liposaccharide (LPS)-induced AKI and found that it can attenuate AKI through: (1) an anti-oxidative mechanism *via* increasing glutathione (GSH), superoxide dismutase (SOD)-1, and SOD-2 and *via* decreasing nitric oxide synthase (iNOS), p47phox, and gp91phox (subunits of renal NADPH oxidase) and (2) an anti-apoptotic mechanism (40).

Du et al. studied the effect of paracalcitol on LPS-AKI and found that its administration ameliorates AKI through: (1) an anti-inflammatory mechanism *via* reduction of TLR-4 and (2) an anti-apoptotic mechanism *via* elevation of Bcl-2 (it is anti-apoptotic) and *via* decrease of caspase-3, PUMA (it is a pro-apoptotic member of the Bcl-2 family), and miR-155 (it targets Bcl-2 and blocks its expression) (41). Regarding the decrease of PUMA and miR-155, it has been found that this is due to the VitD-dependent inhibition of NFκB expression by disrupting the IKK kinase complex or by blocking the p65/p50 nuclear translocation (41).

Park et al. investigated the impact of paricalcitol on cisplatin-induced AKI and found that it ameliorated AKI through: (1) inhibiting EMT, (2) reducing apoptosis *via* increasing Bcl-2 and *via* decreasing P-p53 and p21, p-Bad, Bax, and caspase-3 expression, and (3) increasing the cell proliferation *via* elevating the expression of p27^kip1^ and decreasing that of cyclin-dependent kinase-2 (CDK2) and Cyclin E. The underlying mechanism of these actions includes the inhibition of TGF-β1 and p53 signaling pathways and the elevation of the p27^kip1^ signaling pathway (42).

Another study on the effect of paricalcitol on cisplatin-induced AKI demonstrated that the ameliorated AKI was correlated with reduced lipid peroxidation and ferroptotic cell death due to the direct binding of VDR to the glutathione peroxidase 4 (GPX4) promoter, which induces the expression of GPX4, affecting the induction of ferroptosis (43).

Also, Moneim et al. studied the impact of alfacalcidol and BQ-123, a selective endothelin receptor A blocker (ET_A_R blocker), on cisplatin-induced AKI. Their administration led to attenuation of AKI *via* a VDR-, ET-1-, and ET_A_R-dependent mechanism, which includes the signaling cascade of Pnf-κBp65, TNF-α, and TGF-β1. The combined administration of both drugs led to an enhanced therapeutic effect. These results propose a merger of VitD and endothelin-1 signaling pathways, which is promising as a therapeutic option for cisplatin-induced AKI (44).

In another study, administration of 1,25(OH)_2_D_3_ on gentamicin-induced AKI had no beneficial effect on ameliorating AKI (45). However, there was a decrease of the systolic blood pressure, to some extent, and an increase of the urine volume, probably due to its inhibitory role in RAAS. Also, VitD acted as an antioxidant factor by increasing GSH (45). So, VitD may be a promising therapeutic agent due to its RAAS- and GSH-related action.

Additionally, El-Boshy et al. studied the role of VitD in paracetamol-induced AKI and liver failure. They found that both the therapeutic and the prophylactic use of VitD can ameliorate AKI and liver failure through: (1) an anti-apoptotic mechanism *via* reducing caspase-3 and (2) a decrease of Cyp24α1 and VDBP expression and an increase of Cyp27b1, Cyp2R1, and VDR expression, (3) an anti-oxidative and anti-inflammatory mechanism *via* decreasing IL1-β, IL1R1 (IL-1 receptor 1), IL-6, IL6R (IL-6 receptor), IFN-γ, IFNGR1 (IFN-γ receptor 1), IL17A, and IL17RA (IL-17 receptor A) and *via* elevating GSH, chloramphenicol acetyltransferase (CAT), Gpx, IL10 (and its gene), IL22, and IL22RA (IL-22 receptor A) (46). The effect of the prophylactic use of VitD has been found to be greater than that of its use for treatment (46).

In another study, the administration of 500 and 1,000 IU/kg VitD can ameliorate AKI and liver failure induced by paracetamol. Interestingly, the dose of 500 IU/kg presented a greater protective role (47). The underlying mechanism includes: (1) an anti-oxidative action *via* the reduction of the expression of heme oxygenase 1 (HO-1) and its regulators, NrF2 and BACH1, and (2) an anti-inflammatory action *via* the reduction of NFκB, TNF-α, and IL-10 (47).

Finally, Reis et al. studied the impact of calcitriol on rhabdomyolysis-induced AKI and found that AKI was ameliorated through: (1) an anti-inflammatory mechanism *via* decreasing NFκB and Jun N-terminal kinase (p-JNK), MCP-1, and IL-1β, (2) an anti-oxidative mechanism *via* elevating SOD and *via* decreasing 8-epi-PGF2α and nitrotyrosine, (3) reducing vimentin, proliferating cell nuclear antigen (PCNA), and caspase-3, and (4) elevating CYP24 (48).

### Vitamin D Toxicity Induced AKI

Although VitD seems to ameliorate AKI, its administration should be performed with extreme caution. Hypervitaminosis D can significantly afflict kidney function by inducing hypercalcemia and hyperphosphatemia. Specifically, hypercalcemia leads to the development of nephrogenic diabetes insipidus, which affects water homeostasis by causing polyuria and diuresis. Therefore, there is evident loss of water, leading to hypovolemia, which causes AKI and concomitantly deteriorates the hypercalcemia. So, there is a continuous circle of hypercalcemia causing hypovolemia-induced AKI and vice versa (49–51). Moreover, hypercalcemia and hypercalciuria can lead to deposition of calcium, thus causing nephrolithiasis and renal calcification, which can cause the development of AKI (49–51). Also, hypercalcemia can cause renal vasoconstriction, which subsequently leads to severe GFR decrease and AKI (49–51). As for the hyperphosphatemia, it can cause acute phosphate nephropathy due to the tubulointerstitial deposition of phosphate calcium, a condition that concomitantly worsens the already-existing hyperphosphatemia, thus leading to a vicious circle of deterioration (49–51). As for the acute phosphate nephropathy, it can also be due to significant phosphate intake and the subsequent diarrhea-induced hypovolemia (52). Specifically, the hypovolemia leads to great reabsorption of water and sodium chloride in the proximal tubule and the descending limb of Henle's loop, while calcium and phosphate are not that easily reabsorbed (52). As a result, the greater concentration of these substances in the distal tubule and the collecting duct leads to significant deposition of calcium phosphate in these structures, a deposition that is exacerbated due to surface expression of hyaluronan and osteopontin as a result of hypovolemia-induced tubular injury (52). Among risk factors for the development of acute phosphate nephropathy are: advanced age, hypertension, CKD, drugs (such as angiotensin-converting enzyme inhibitors, angiotensin II receptor blockers, and loop- or thiazide-type diuretics), female gender, diabetes mellitus, and the use of non-steroidal anti-inflammatory drugs (NSAIDs) (52). As for the treatment of acute phosphate nephropathy, kidney replacement therapy (KRT) can be used to tackle this condition, and particularly, sustained low efficiency dialysis (SLED) has gained ground in treatment, given that it is a hybrid technique, which combines the advantages of both intermittent and continuous KRT (53).

VitD-mediated hypercalcemia can be due to: (1) excessive VitD_2_ or D_3_ ingestion/supplementation, (2) extravagant calcitriol ingestion or pharmaceutical administration, (3) elevated ectopic production of calcitriol (evident in granulomatous diseases, such as tuberculosis, sarcoidosis, leprosy, fungal infections, and others; in Hodgkin's and non-Hodgkin's lymphomas; in malignant lymphoproliferative diseases), (4) milk-alkali syndrome (MAS), and (5) depleted catabolism of calcitriol due to mutations of CYP24A1 genes (54).

VitD toxicity due to immoderate administration of VitD supplements or overfortified milk is a global phenomenon (55), potentially affecting kidney function. Chowdry et al. presented a study of VitD toxicity incidents in a tertiary care center at the Sher-i-Kashmir Institute of Medical Sciences, in which 16 out of 19 patients where identified with hypervitaminosis D–induced AKI due to extravagant doses of VitD (median cumulative dose of VitD is 6,000,000 IU), in order to correct VitD deficiency. Not all the patients with toxic levels of 25(OH)D (>150 ng/ml) developed symptoms (56). Similarly, 13 patients in Brazil developed AKI due to intramuscular injection of veterinary supplements of vitamins A, D, and E for esthetic purposes (57).

Also, VitD toxicity–induced AKI has been observed due to anabolic steroid and VitD supplement abuse (58), dispensing errors (59), dosage malpractice and overcorrection of VitD deficiency (60), intramuscular injection of VitD after operation (61), overfortification of milk with VitD (62), over-the-counter supplements (63), and topical treatment with calcitriol analog in combination with oral calcium/VitD for psoriasis (64).

Additionally, in another study, 33 patients with osteoporosis treated with 0.75 μg/day eldecalcitol developed hypercalcemia-induced AKI since the discontinuation of eldecalcitol ameliorated the situation (65). Furthermore, 11 patients developed with hypercalcemia-induced AKI, due to alfacalcidol (9 patients) or calcitriol (2 patients) (65).

Moreover, VitD toxicity–induced AKI is related with granulomatous diseases (especially sarcoidosis), MAS, subclinical hyperparathyroidism, and immune reconstitution syndrome.

Tollitt and Solomon presented a case of a 38-year-old male and two other patients with a history of sarcoidosis. After treatment with high doses of cholecalciferol, the patient developed different symptoms of hypercalcemia, such as vomiting, nausea, muscle cramps, and constipation, and subsequent AKI (66). In these cases, the hypercalcemia is due to the extrarenal synthesis of 1,25(OH)_2_D by macrophages within the sarcoid granulomas, the process of which is without systemic regulation (67, 68). The sufficient administration of prednisolone inhibits macrophages' 1-α hydroxylase activity and protects against hypercalcemia (66). A similar relation between VitD administration and VitD-induced hypercalcemia has been observed in tuberculosis and Hodgkin's lymphomas (69, 70).

MAS is a result of VitD, calcium carbonate, and bisphosphonate administration for osteoporosis, iatrogenic hypothyroidism, and idiopathic hypothyroidism treatment. Although there is limited knowledge regarding its pathophysiology, it affects patients who absorb more calcium than average. Among the risk factors are old age, medication that reduces GFR, and hypovolemia. MAS consists of hypercalcemia, metabolic alkalosis, and renal function disruption, and, particularly, hypercalcemia leads to AKI *via* renal vasoconstriction, polyuria, and GFR depletion (71, 72).

Asghar et al. presented a case of a 55-year-old female who had slightly elevated calcium and was found to be VitD-deficient. The prescription of VitD led later to the development of a giant cystic parathyroid adenoma and to manifestation of parathyroid crisis, accompanied by severe gastrointestinal symptoms and AKI. This suggests that VitD administration can unveil subclinical hyperparathyroidism (73).

Finally, Tsao et al. described a case of immune reconstitution inflammatory syndrome (IRIS) due to *Mycobacterium tuberculosis* lymphadenitis. It was manifested in a patient with history of silent human immunodeficiency virus-1 (HIV-1) infection, who was in highly active antiretroviral therapy (HAART) because of plasma viral load increase. This 48-year-old male patient, after the initiation of HAART, developed various hypercalcemia symptoms, accompanied by hypervitaminosis D [1,25(OH)_2_D_3_, 268 pmol/L], which progressed to coma and AKI. IRIS can be due to many opportunistic infections, but the most common infection is *M. tuberculosis*, which is incriminated as the cause of VitD-induced hypercalcemia, which leads to AKI (74). All cases of hypervitaminosis D–induced AKI are summarized in Table 3.

**Table 3:** Cases of hypervitaminosis D as cause of AKI.

**References**	**Article type**	**Sample size**	**Substance**	**Concentration**
Chowdry et al. (56)	Case series	19 patients	25(OH)D	371 (190–988) ng/ml
Daher et al. (57)	Case series	16 patients	Vitamin D	135 ± 75 ng/ml
Daher et al. (58)	Case series	2 patients	Vitamin D	N/A
Nasri et al. (59)	Case	1 patient	Vitamin D	>400 nmol/L
Kaur et al. (60)	Case series	16 patients	Serum 25(OH)D	371 (175–1,161) ng/ml
Bansal et al. (61)	Case	1 patient	Serum 25(OH)D	150 ng/ml
Jacobus et al. (62)	Case series	8 patients	Serum 25(OH)D	293 ± 174 ng/ml
Koutkia et al. (63)	Case	1 patient	Serum 25(OH)D	487.3 ng/ml
Corden et al. (64)	Case	1 patient	Vitamin D	N/A
Aihara et al. (65)	Case series	43 patients	Eldecalcitol (32 patients) Alfacalcidol (9 patients) Calcitriol (2 patients)	N/A
Tollit et al. (66)	Case	1 patient	Vitamin D	N/A
Lavender et al. (69)	Case	1 patient	1,25(OH)2D_3_	318 pmol/L
Karmali et al. (70)	Case	1 patient	1,25(OH)2D_3_	145 pg/ml
Altun et al. (71)	Case	1 patient	Calcitriol	N/A
Jeong et al. (72)	Case	1 patient	Calcitriol	N/A
Asghar et al. (73)	Case	1 patient	Vitamin D	119 ng/ml
Tsao et al. (74)	Case	1 patient	1,25(OH)2D_3_	268 pmol/L

*This table presents the different studies that have been done regarding vitamin D toxicity as a cause of AKI. It depicts information regarding the type of study conducted, the sample size, the vitamin D–related substance that has been calculated, and the concentration of this substance*.

## Conclusion

It can be concluded that there is a two-way relation between AKI and VitD. Specifically, disruptions of VitD—both hypovitaminosis and hypervitaminosis—can lead to the development of AKI, while also, AKI can contribute to dysregulation of VitD's homeostasis and function.

On this ground, due to this two-way causality relation, VitD is examined on whether its forms can act as a novel biomarker of AKI. Many studies have confirmed VitD's significant role as a predictive (it can help to determine the risk of developing AKI) and prognostic (it can help determine the stage, progress, clinical outcomes, and mortality risk of AKI) biomarker.

Finally, VitD is found to be a potentially important therapeutic factor for AKI due to its multisystemic functions, which include regulation of many enzymic mechanisms *via* genomic and non-genomic actions.

However, many prospective studies and trials need to be conducted, in order to: (1) fully determine the complex relation between VitD and AKI, (2) find the best cutoff points with the most significant statistic importance, and (3) determine the therapeutic protocols of VitD as treatment of AKI and its potential adverse effects.

Despite the necessity of future studies, VitD is a very promising biomarker and a potential treatment for AKI, which is one the most prominent health problems nowadays.

## Data Availability Statement

The original contributions generated in the study are included in the article/Supplementary Material, further inquiries can be directed to the corresponding author.

## Author Contributions

MP and TP: conceptualization and supervision. SG and MP: data curation and investigation. SG: formal analysis and writing—original draft. All authors methodology and writing—review and editing.

## Conflict of Interest

The authors declare that the research was conducted in the absence of any commercial or financial relationships that could be construed as a potential conflict of interest.
