# Supplementary material for: Vitamin D and Acute Kidney Injury: A Two-Way Causality Relation and a Predictive, Prognostic, and Therapeutic Role of Vitamin D
Source: Front Nutr. 2021 Mar 4;7:630951. doi: 10.3389/fnut.2020.630951 (PMC7969500; doi:10.3389/fnut.2020.630951)
Supplement: Supplementary file 1 [file Table_1.docx]

***Supplementary Table 1.* Vitamin D and its analogs as biomarkers for AKI’s prediction and prognosis.** This table presents the results of each study regarding the use of vitamin D and its metabolites both as predictive (prediction of AKI) and as prognostic (regarding the progression of AKI, the risk of mortality and morbidity such as sepsis, MARE, hospitalization etc.; AKI=acute kidney injury, OR= odds ratio; Adjusted OR= odds ratio after multivariable adjustments; 95% CI= 95% confidence interval; hCAP-18= human cathelicidin antimicrobial peptide-18

a= adjusted for age, gender, race (white, nonwhite), Deyo-Charlson index, sepsis, and type (surgical vs. medical)

b= adjusted for age, gender, race (white, nonwhite), Deyo-Charlson index, season, and type (surgical vs. medical)

c= adjusted for age, admission SCr

d= adjusted for age, admission SCr, admission albumin, VDBP

e= adjusted for presence of heart failure, total volume of contrast medium used, hemoglobin levels

f= adjusted for sex, race, diabetes mellitus, eGFR, urine albumin-creatinine ratio

g= adjusted for sex, race, diabetes mellitus, eGFR, urine albumin-creatinine ratio, age, hypertension status, education

h= adjusted for sex, race, diabetes mellitus, eGFR, urine albumin-creatinine ratio, age, hypertension status, education, calcium, phosphorus, PTH

i= adjusted for age and APACHE II score

| **Study** | **Article type** | **Sample size** | **Biomarker used** | **Predictive Outcome** | | **Prognostic Outcome** | |
| --- | --- | --- | --- | --- | --- | --- | --- |
| Braun et al. (25) | Two-center observational study | 2075 patients, aged ≥18 years old | Serum 25(OH)D | **Deficiency levels (<15ng/ml)** | **Prediction of AKI**  OR=1.72  (95% CI=1.42–2.02; p<0.0001)  Adjusted OR^a^=1.41  (95% CI=1.04–1.92; p<0.03) | **Deficiency levels (<15ng/ml)** | **30-day mortality**  OR=1.60  (95% CI=1.18–2.17; p=0.003)  Adjusted OR^b^=1.61  (95% CI=1.06–1.57; p=0.004) |
|  |  |  |  | **Insufficiency levels**  **(15-30ng/ml)** | **Prediction of AKI**  OR=1.27  (95% CI=1.03–1.58; p=0.03)  Adjusted OR^a^=1.43  (95% CI=1.08–1.88; p=0.01) | **Insuffiency levels**  **(15-30ng/ml)** | **30-day mortality**  OR=1.35  (95% CI=1.02–1.79; p=0.04)  Adjusted OR^b^=1.41  (95%=1.05–1.21; p=0.02) |
| Zapatero et al. (26) | Single-center, prospective observational study | 135 patients admitted in the ICU | Serum 25(OH)D | **Prediction of AKI**  Higher rates of AKI in patients with vitamin D <10,9 ng/ml (29%) than in patients with vitamin D sufficiency (13%); p=0.02 | | **Mortality**  OR=2.86 (95% CI=1.05-7.86; p=0.04)  AUC=0.61 (95% CI=0.51-0.75)  Best cut-off=10.9 ng/ml  Lower 25(OH)D in non-survivor patients (8.14[6.17-11.53]ng/ml) than survivor patients (12 [7.1-20.30] ng/ml); p=0.04 | |
| Leaf et al. (27) | Prospective cohort study | 60 participants (30 participants with AKI and 30 controls) | Bioavailable 25(OH)D | **No significant association** | | **Severity of sepsis**  Negative correlation with r= -0.45; p <0.001 | |
|  |  |  |  |  |  | **Mortality**  Lower admission levels in non-survivor group [0.5 (0.4-0.8) ng/ml] than in the survivor group [1.2 (0.6-2.0) ng/ml]; p= 0.05  Adjusted OR_1_^c^= 0.16 (95% CI=0.03-0.85)  Adjusted OR_2_^d^= 0.05 (95% CI=0.004-0.64) | |
|  |  |  | 25(ΟΗ)D | **No significant association** | | **Severity of sepsis**  Negative correlation with r= -0.42; p= 0.001 | |
|  |  |  | VDBP | **Prediction of AKI**  Lower admission levels in AKI (23 [15-31] mg/dl) than in control group (29 [25–36] mg/dl), p=0.003 | | **No significant association** | |
|  |  |  | 1,25(OH)_2_D | **Prediction of AKI**  Lower admission levels in AKI (17 [10–22] pg/ml) than in control group (25 [15–35] pg/ml); p=0.01 | | **No significant association** | |
|  |  |  | FGF-23 | **Prediction of AKI**  Higher admission levels in AKI (1471 [224–2534] RU/ml) than in control group (263 [96–574] RU/ml); p=0.003 | | **Severity of sepsis**  Positive correlation with r= 0.35; p=0.007 | |
| Sahin et al. (28) | Prospective study | 403 patients | Vitamin D | **Prediction of CIN-AKI**  Lower levels of vitamin D in the CIN-AKI group (8.5 [0.5-26.6] ng/ml) than in non-CIN-AKI group (14.9 [1.9-93.5] ng/ml); p=0.01  OR= 1.17 (95% CI=1.10-1.23; p=0.01)  Adjusted OR^e^= 1.18 (95% CI=1.11-1.26; p=0.01) | | **N/A** | |
| Vicente-Vicente et al. (30) | Rat model | 48 rats | uVDBP | **Prediction of risk of gentamicin-induced AKI**  Elevated levels of uVDBP associated with chronic sensitization-proclivity to gentamicin nephrotoxicity | | **N/A** | |
| Rebholz et al. (31) | Nested case-control study | 435patients (184 ESRD patients, 251 control patients) | VDBP | **N/A** | | **Development of ESRD stage RIFLE-AKI**  Higher levels of in ESRD-patients (260 [233-288] μg/ml) than control group (250 [225,277] μg/ml); p=0.02  Adjusted OR_1_^f^=1.48  (95% CI=1.08-2.02; p=0.02)  Adjusted OR_2_^g^=1.41  (95%CI=1.02-1.95; p=0.04)  Adjusted OR_3_^h^=1.76  (95% CI=1.22-2.54; p=0.003) | |
|  |  |  | Free 25(OH)D | **N/A** | | **Development of ESRD stage RIFLE-AKI**  Lower levels in ESRD-patients (6.79 [4.91-9.44] pg/ml) than control group (8.08 [5.66-11.21] pg/ml); p=0.003  Adjusted OR_1_^f^=0.64  (95% CI=0.47-0.89; p=0.007)  Adjusted OR_2_^g^=0.64  (95% CI=0.46-0.89; p=0.009)  Adjusted OR_3_^h^=0.65  (95% CI=0.46-0.92; p=0.2) | |
|  |  |  | Bioavailable 25(OH)D | **N/A** | | **Development of ESRD stage RIFLE-AKI**  Lower levels in ESRD-patients (2.21 [1.63-3.15] ng/ml) than control group (2.69 [1.83-3.79] ng/ml); p=0.001  Adjusted OR_1_^f^=0.63  (95% CI=0.44-0.88; p=0.007)  Adjusted OR_2_^g^=0.63  (95% CI=0.44-0.90; p=0.01)  Adjusted OR_3_^h^=0.63  (95% CI=0.43-0.91; p=0.02) | |
|  |  |  | Detectable 3-*epi*-25(OH)D | **N/A** | | **No significant association** | |
|  |  |  | 25(OH)D | **N/A** | | **Development of ESRD stage RIFLE-AKI**  Lower levels in ESRD-patients (18.6 [13.5-25.7] ng/ml) than control group (20.3 p15-27.3] ng/ml); p=0.04  Adjusted OR_1_^f^=0.7  (95% CI=0.51-0.97; p=0.03)  Adjusted OR_2_^g^=0.73  (95% CI=0.53-1; p=0.05)  Adjusted OR_3_^h^=0.83  (95% CI=0.58-1.19; p=0.3) | |
|  |  |  | 1,25(OH)_2_D | **N/A** | | **Development of ESRD stage RIFLE-AKI**  Lower levels in ESRD-patients (34 [26-45.6] pg/ml) than in control group (39 [30.4-50.4] pg/ml); p=0.001  Adjusted OR_1_^f^=0.87  (95% CI=0.61-1.26; p=0.5)  Adjusted OR_2_^g^=0.82  (95% CI=0.56-1.2; p=0.3)  Adjusted OR_3_^h^=0.73  (95% CI=0.0.48-1.13; p=0.2) | |
|  |  |  | Deficient 25(OH)D (<30ng/ml) | **N/A** | | **No significant association** | |
| Chaykovska et al. (29) | Prospective cohort study | 314 patients with diabetes melitus or mild renal impairment | uVDBP | **No significant association** | | **Need of dialysis**  Higher levels in dialysis group (613.07±700.45ng/ml) than in no-dialysis group (113.06±299.61ng/ml); p<0.001 | |
|  |  |  |  |  |  | **Mortality**  Higher levels in non-survivor group (522.01±521.86 ng/ml) than in survivor group (121.41±324.45ng/ml); p=0.04 | |
|  |  |  |  |  |  | **MARE**  Higher levels in MARE group (506.16±624.61ng/ml) than in no-MARE group (112.08±302.00ng/ml); p<0.001 | |
|  |  |  |  |  |  | **Non-elective Hospitalization**  Higher levels in non-elective hospitalization group (291.77±573.39ng/ml) than in no-non-elective group (102.81±262.81ng/ml); p=0.001 | |
|  |  |  | uVDBP/uCr | **No significant association** | | **Need of dialysis**  Higher levels in dialysis group (169±244.52ng/ml) than in no-dialysis group (16.02±48.05ng/ml); p<0.001 | |
|  |  |  |  |  |  | **Mortality**  Higher levels in non-survivor group (139.57±222.29ng/ml) than in survivor group (18.62±61.09ng/ml); p<0.001 | |
|  |  |  |  |  |  | **Non-elective Hospitalization**  Higher levels in non-elective hospitalization group (54.01±130.36ng/ml) than in no-non-elective group (16.07±54.23ng/ml); p=0.002 | |
| Lai et al. (32) | Prospective cohort study | 200 patients | 1,25(OH)_2_D | **Prediction of AKI**  Lower levels in AKI-group (59.6±53.0 pmol/l) than in healthy-group (86.2±35.3 pmol/l) and in critically-without-AKI-group (98.8±39.7 pmol/L); p=0.006 | | **AKI-stages stratification**  Negative correlation between its levels and Risk (72.6±69.4pmol/l), Injury (53.7±32.7pmol/l) and Failure (42.2±29.3pmol/l) stages of AKI | |
|  |  |  |  |  |  | **Mortality**  **No significant association** | |
|  |  |  | 25(ΟΗ)D | **No significant association** | | **No significant association** | |
|  |  |  | [1,25(OH)_2_D/ 25(OH)D] x1000 | **Prediction of AKI**  Lower levels in AKI-group (1.81±1.49) than in healthy-group (3.61±2.28) and in critically-without-AKI-group (3.17±1.23); p <0.001 | | **AKI-stages stratification**  Negative correlation between its levels and Risk (2.24±1.78), Injury (1.65±1.36) and Failure (1.39±0.95) stages of AKI | |
|  |  |  |  |  |  | **Mortality**  **No significant association** | |
| Leaf et al. (33) | Prospective cohort study | 121 critically ill ICU patients | hCAP-18 (on ICU day 1) | **No significant association** | | **Sepsis**  OR (tertile1 vs tertile 3) = 2.35  (95% CI=0.96-5.74; p=0.06)  Adjusted OR^i^ (tertile1 vs tertile 3) = 2.54  (95% CI=1.01-6.4; p=0.047)  OR (tertile2 vs tertile3) = 3.72  (95% CI=1.47-9.45; p=0.006)  Adjusted OR^i^ (tertile2 vs tertile 3) = 3.64  (95% CI= 1.4-9.44; p=0.008) | |
|  |  |  |  |  |  | **90-day mortality**  Lower levels in non-survivor (116 [88-178] ng/ml) than in survivor group (176 [112-304] ng/ml); p=0.04  OR (tertile1 vs tertile3) = 4.8  (95% CI=1.23-18.8; p=0.02)  Adjusted OR^i^ (tertile1 vs tertile3) = 4.49  (95% CI=1.08-18.67; p=0.04) | |
|  |  |  | Free 25(OH)D (on ICU day 3) | **N/A** | | **90-day mortality**  Lower levels in non-survivor (7.7 [5.9-9.3] ng/ml) than in survivor group (9.9 [7-13.1] ng/ml); p=0.03 | |
|  |  |  | Bioavailable 25(OH)D (on ICU day 3) | **N/A** | | **No significant association** | |
