# Supplementary material for: Vitamin D and Acute Kidney Injury: A Two-Way Causality Relation and a Predictive, Prognostic, and Therapeutic Role of Vitamin D
Source: Front Nutr. 2021 Mar 4;7:630951. doi: 10.3389/fnut.2020.630951 (PMC7969500; doi:10.3389/fnut.2020.630951)
Supplement: Supplementary file 2 [file Table_2.docx]

***Supplementary Table 2.* Vitamin D as a potential treatment of AKI and the mechanism of action.** This table presents the therapeutic effects of vitamin D and its analogues in different animal models of AKI. It, also, depicts the underlying mechanism of vitamin D’s ameliorating action regarding AKI; MMP= matrix metalloproteinase; NHE-1= Na+/H+ exchanger-1; TLR-4= Toll-like receptor-4; IFN-γ= interferon-γ; IGFBP-7= Insulin-like growth factor binding protein-7; TIMP-2= tissue inhibitor metalloproteinase-2; CCL= creatinine clearance; PPAR-γ= peroxisome proliferator-activated receptor-γ ; FeNA= fractional excretion of sodium; MPO= myeloperoxidase; TBARS= thiobarbituric acid reactive substances; GSH= glutathione; CAT= chloramphenicol acetyltransferase; CD68= cluster of differentiation-68; MCP-1= monocyte chemoattractant protein-1; sCr= serum creatinine; BUN= blood urea nitrogen; NGAL= neutrophil gelatinase-associated lipocalin; PAS= periodic acid-Schiff; TUNEL= terminal deoxynucleotidyl transferase dUTP nick end labeling; SOD-1= superoxide dismutase-1; SOD-2= superoxide dismutase-2; iNOS= inducible nitric oxide synthase; TNF= tumor necrosis factor; VDR= VitD receptor; α-SMA= α-smooth muscle actin; ERK1/2= extracellular signal-regulated kinase-1/2; CTGF= connective tissue growth factor; CDK2= cyclin-dependent kinase-2; PCNA= proliferating cell nuclear antigen; MDA= malondialdehyde; 4HNE= 4-Hydroxy-2-Nonenal; GPX4= glutathione peroxidase 4; ET-1= endothelin-1; ETAR= endothelin receptor A; ETBR= endothelin receptor B; KIM-1= kidney injury molecule-1; GGT= γ-glutamyl-transferase; RAAS= renin-angiotensin=aldosterone system; ALT= alanine transaminase; AST= aspartate transaminase; ALP= alkaline phosphatase; VDBP= VitD binding protein; IL-1β= interleukin-1β; IL-6= interleukin-6; IL-10= interleukin-10; IL-22=interleukin-22; IL22RA= interleukin-22 receptor A; IL-17= interleukin-17; p-JNK=phosphorylated c-Jun N-terminal kinase; IFNGR1= interferon-γ receptor-1; IL6R= interleukin-6 receptor; IL17RA= interleukin-17 receptor-A; NrF2= nuclear factor erythroid 2–related factor 2; BACH1= BTB and CNC homology 1; CK= creatine kinase; 8-epi-PGF2= 8-epi-placental growth factor

| **Author** | **Animal-Sample size** | **Treatment used** | **Result** | **Utility** |
| --- | --- | --- | --- | --- |
| Ersan et al. (34) | 20 Wistar rats | Paracalcitol | Decreased level and expression of MMPs | IRI |
|  |  |  | Decreased tubular injury scores |  |
| Hamzawy et al (35) | 30 Wistar rats | OCT | Decrease of NHE-1 exchanger, TLR-4, IFN-γ, Bax/Bcl-2, cytochrome c and caspase-3, IGFBP-7, TIMP-2, serum 25(OH)D and CCL |  |
|  |  |  | Increase of Beclin-1 and LC3II/LC3I ratio |  |
| Kapil et al (36) | 48 Wistar rats | Vitamin D | PPAR-γ-mediated improvement of CCL, urea, uric acid, potassium, calcium, microproteinuria, FeNa, MPO, TBARS, GSH and CAT |  |
| Arfian et al. (37) | 20 Swiss Webster mice | Vitamin D | Decrease of tubular injury score, α-SMA (marker of myofibroblast), CD68 (marker of macrophages), TLR4, MCP1 |  |
| Silva Barbosa et al. (38) | C57BL/6 mice | Inhibition of Sult1e1 (it affects vitamin D) | Decrease of sCr, BUN, NGAL (marker of renal injury), PAS (tubular injury marker), TUNEL (apoptosis marker), renal and liver IL-6, Fgg gene |  |
|  |  |  | Increase of Cyp24α1 and Ccnd1 genes |  |
| Lee et al. (39) | 70 C57BL/6 mice | Paracalcitol | TLR4-NF-κB mediated increase of cytoplasmic IκB |  |
|  |  |  | TLR4-NF-κB mediated decrease of histological damage, BUN, TUNEL^+^ cells, neutrophil- and macrophage-related infiltration, IL-6, MCP-1 |  |
| Xu et al. (40) | 96 CD-1 mice | Vitamin D_3_ | Increase of GSH, SOD-1, SOD-2 | LPS-induced AKI |
|  |  |  | Decrease of histological damages, BUN, uric acid, albumin, renal protein nitration, renal p47phox and gp91phox, renal peroxidation, TUNEL, iNOS |  |
| Du et al. (41) | C57BL/6 mice | Paracalcitol | Decrease of body weight loss, BUN, SCr, TNF-α, IL-1β, IL-6, and MCP-1, TUNEL, caspase-3, PUMA, miR-155, |  |
|  |  |  | Increase of VDR, Bcl-2 |  |
| Park et al. (42) | 36 Sprague–Dawley rats | Paracalcitol | Decrease of α-SMA, Smad-2/3, Smad-4, TGF-β, p21, P-53, p-Bad, TUNEL^+^ cells, ERK1/2 and P-p38, fibronectin, CTGF, total/cleaved caspase-3, p27kip1, CDK2, cyclin E, Bax and proliferating cell nuclear antigen (PCNA) | Cisplatin-induced AKI |
|  |  |  | Increase of E-cadherin and Bcl-2 |  |
| Hu et al. (43) | 72 male C57BL/6 mice | Paricalcitol | Decrease of serum BUN, SCr, renal histological injury, cell death, 4HNE (marker of lipid peroxidation), mitochondrial injury, MDA (marker of lipid peroxidation), erastin (marker of ferroptosis) |  |
|  |  |  | Increase of GPX4 |  |
| Moneim et al. (44) | 128 male Sprague–  Dawley rats | Alfacalcidol | Decrease of SCr, serum urea, ET-1, ET_A_R, pNFκBp65, TNF-α, TGF-β1, ATN and renal histopathological scores |  |
|  |  |  | Increase of ET_B_R, VDR |  |
|  |  | BQ-123 | Decrease of SCr, serum urea, ET-1, ET_A_R, pNF-κBp65, TNF-α, TGF-β1, ATN and renal histopathological scores |  |
|  |  |  | Increase of VDR |  |
|  |  | Alfacalcidol + BQ-123 | Enhanced (in comparison with the use of each drug alone) decrease of SCr, serum urea, ET-1, ET_A_R, pNF-κBp65, TNF-α, TGF-β1, ATN and renal histopathological scores |  |
|  |  |  | Enhanced (in comparison with the use of each drug alone) increase of ET_B_R, VDR |  |
| Hur et al. (45) | 30 Wistar albino rats | 1,25(OH)D_3_ | No beneficial effect on tubular degeneration, tubular necrosis, tubulointerstitial nephritis and total histological scores, KIM-1, GGT, NGAL | Gentamicin-induced AKI |
|  |  |  | Increase of GSH |  |
|  |  |  | Decrease of systolic blood pressure via RAAS |  |
| El-Boshy et al. (46) | 32 Wistar rats | Treatment with Vitamin D | Decrease of ALT, AST, ALP, serum urea, histological damage, caspase 3, TUNEL, Cyp24α1, VDBP, MDA, IL1β, IL6, IFN-γ, IL17A, IL1R1-gene, IFNGR1-gene, IL6R-gene, IL17RA-gene | Paracetamol-induced AKI (along with liver damage) |
|  |  |  | Increase of serum total protein, serum albumin, total serum 25(OH)D, Cyp2R1, Cyp27b1, VDR, GSH, CAT, Gpx, IL10, IL22, IL10-gene, IL22RA-gene |  |
|  |  | Prophylactic use of Vitamin D | Greater decrease (than the treatment with vitamin D) of ALT, AST, ALP, serum urea, histological damage, caspase 3, TUNEL, Cyp24α1, VDBP, MDA, IL1β, IL6, IFN-γ, IL17A, IL1R1-gene, IFNGR1-gene, IL6R-gene, IL17RA-gene |  |
|  |  |  | Greater increase (than the treatment with vitamin D) of serum total protein, serum albumin, total serum 25(OH)D, Cyp2R1, Cyp27b1, VDR, GSH, CAT, Gpx, IL10, IL22, IL10-gene, IL22RA-gene |  |
| El-Magd et al. (47) | 40 male Sprague-Dawley rats | Vitamin D | Decrease of ALT, AST, SCr, serum urea, MDA, total antioxidant capacity, HO-1, IL-10, TNF-α, liver histopathological score, renal histopathological score, NrF2, BACH1, NFκB |  |
| Reis et al. (48) | 37 Wistar Hannover rats | Calcitriol | Decrease of serum CK, urinary flow, ATN, ED1 (marker of macrophages), NF-Κb and p-JNK, MCP-1, IL-1β, 8-epi-PGF2 α (oxidative marker), nitrotyrosine (oxidative marker), vimentin, PCNA, caspase-3, VDBP | Rhabdomyolysis-induced AKI |
|  |  |  | Increase of EC-SOD, CYP24 |  |
